# A Qualitative Investigation into the Experiences of Students with Developmental Coordination Disorder (DCD/Dyspraxia) in Higher Education

**DOI:** 10.3390/ejihpe14120203

**Published:** 2024-12-20

**Authors:** Judith Gentle, Mirela Ivanova, Marie Martel, Scott Glover, Anesa Hosein

**Affiliations:** 1 School of Psychology, University of Surrey, Guildford GU2 7XH, UKm.martel@surrey.ac.uk (M.M.); 2 Department of Psychology, Royal Holloway University of London, Egham TW20 0EX, UK; scott.glover@rhul.ac.uk

**Keywords:** developmental coordination disorder, dyspraxia, neurodevelopmental conditions, higher education, students, specific learning difficulty

## Abstract

Developmental coordination disorder (DCD/Dyspraxia) is a commonly misunderstood and under-recognized specific learning difficulty (SpLD) in educational settings. This lifelong condition affects fine and gross motor coordination and significantly interferes with many activities of daily living, academic achievement, and employment opportunities. However, most Higher Education Institutions (HEIs) are unaware of its prevalence within their context, even though 5% of the general population have DCD and the enrolment of students in UK Higher Education with a known disability has increased by 42.4% between 2018 and 2023. Thus, understanding the lived experiences of students with DCD within Higher Education in the UK remains a considerable gap in knowledge. Through the use of focus groups, the lived experiences of 10 students with DCD at two UK HEIs were investigated. The thematic analysis identified four main themes: ‘Awareness of DCD’, ‘Participation in Higher Education for individuals with DCD’, ‘Wellbeing’, and ‘Everyday living’. Students shared that HEIs appeared to lack awareness of DCD and felt they had an inability to specify the correct support at university. Importantly, whilst the students in the study were not always confident in identifying the specific support they needed, they shared the strategies they used to aid their university experience. The students described the physical toll that many everyday living tasks can take, which subsequently negatively impact academic participation and wellbeing. On a positive note, many of the students discussed positive experiences at university, such as enjoying their own autonomy (and flexibility) to be independent and inform strategies for their own learning. Importantly, the findings from this work highlight the complexity and heterogeneity of DCD and the need for a tailored approach to supporting individuals with this condition. Given the importance of educational qualifications to enter the workplace, and the contribution of employment to quality of life, these findings help signpost areas where HEIs can improve the experiences of students with DCD that may also enhance academic success.

## 1. Introduction

Higher education systems have to be socially just, that is, they need to be fair and equitable for all students [1]. Much attention on social justice has focused on areas of inclusion for gender and race. However, we argue that Higher Education Institutions need to adopt the same inclusive rigour for students with specific learning disabilities (conditions that affect a person’s ability to read, write, listen, speak, reason, or do maths) as they do to other areas of inclusion. Higher Education Institutions must ensure that reasonable adjustments are tailored appropriately to promote social justice and enable equal opportunities for success for all students. This is essential not only to fulfil their moral responsibilities, but in some countries, their legal responsibilities as well. This research uses the UK as a case study, where there are legal obligations under the UK’s Equality Act (2010) to ensure there is no discrimination based on disabilities. The aim is to understand the extent of support for students with specific learning difficulties within this legal framework (see ref. [2] for details).

Student enrolment to attend UK universities with a known disability (which includes those with specific learning difficulties) increased by 42.4% between the academic years of 2018/19 and 2022/23 [3]. In this paper, specific learning difficulties refer to conditions such as dyslexia, autism spectrum disorder (ASD), attention deficit hyperactivity disorder (ADHD), and developmental coordination disorder (DCD/dyspraxia). In the UK, the Equality Act (2010) requires educational institutions to provide reasonable adjustments or support for students to reduce any disadvantages [4]. Hence, whilst inclusion is a high priority in UK Higher Education from a legal perspective, the basics of provision for students with specific learning difficulties are often not fully met. This may be due to lack of knowledge, insufficient training, limited time, or even stigmatization associated with disclosure [5,6]. Additionally, different specific learning difficulty profiles are often in receipt of similar types of support, which often include extra time in exams and the provision of laptops [7,8]. However, aetiologies and manifestations of specific learning difficulties differ considerably, and these adjustments may not always be the best option for the students. Sumner et al. [9] argue that an individualised approach is necessary to fully support the student’s needs but note that this creates more complexity for Higher Education Institutions when delivering effective support for students with specific learning difficulties. It is therefore unsurprising that students with specific learning difficulties, who lack individualised support, are more likely to withdraw from their course before completion [10]. Those who do finish achieve lower degree results and have worse employment outcomes than their neurotypical (NT) peers [11,12,13,14]. This is troubling as it creates unfair outcomes and educational inequalities for those with specific learning difficulties.

Within UK Higher Education, great strides have been made to provide appropriate adjustments for students with dyslexia, ASD, and ADHD [15,16,17]. However, students with DCD may receive more uneven support, as education or health professionals tend to be unfamiliar with the appropriate adjustments and support needed for DCD [18,19,20]. The educational inequalities and unfairness of outcomes for these students allow us to question the social justice for students with DCD.

With a prevalence rate of 5–6% [21], which is similar to that of dyslexia and far higher than ASD (at 1–2% [21]), DCD is a common, yet under-recognised, lifelong neurodevelopmental movement coordination condition [18,22]. In DCD, the movement skills are below what would be expected given the person’s age and their opportunity to practice and they significantly impact everyday activities, academic achievement, wellbeing, and employment opportunities [21,23,24,25,26]. Barriers to inclusive practice for university students with DCD are exacerbated by a paucity of research, tortuous pathways to diagnosis, and poor understanding among professionals and the general public [8,27,28,29,30,31]. For example, Kirby et al. [29] reported that students with DCD in the UK were less likely to receive a disability student allowance (DSA; government grant available to students in England and Wales, with similar funding available in Scotland and Northern Ireland) than dyslexic students, despite this group reporting more difficulties in Higher Education. Whilst research by Sumner et al. [9] suggests the differences between students with dyslexia and DCD receiving DSA may be narrowing (dyslexia—66%; DCD—54%), the analysis of the differing sample demographics between these studies may suggest otherwise. For example, the Sumner et al. [9] study invites only those with a diagnosis to participate, whereas the Kirby et al. study [29] invites students to participate in the study if they were ‘clumsy as a child’ and/or had a diagnosis of DCD. Given the chronic underdiagnosis of DCD [29], as evidenced in the Sumner et al. study [9] with very unequal groups (dyslexia, 163; DCD, 50), we suspect many students with DCD are still not being diagnosed, and consequently, they are not receiving DSA. Additionally, reports from students with DCD who do receive support suggest that they find current inclusive teaching practices (larger font on slides, adjustments for spelling mistakes) less helpful and are less likely to access technology-related support (funded laptops, recording devices) than students with dyslexia [9]. Students with DCD are also less likely to choose courses in physical science or health courses, possibly illustrating a lack of desire to engage with disciplines requiring good motor skills [8].

Another barrier to inclusive practice is that DCD is a complex condition [32,33] and the interactions between the primary motor deficits, secondary effects on cognitive systems (such as executive functions), and psychological self-efficacy are not well understood [34,35,36]. Students with DCD experience a wide range of difficulties, such as handwriting (clarity and speed; copying notes at speed), working memory (planning, organisation, time management, prioritising tasks), fatigue, and poor perceptions of their academic capabilities [8,37,38,39,40,41,42], all of which are known to affect academic achievement in Higher Education [43,44].

Kirby et al. [8] found that over 50% of participants with DCD specified that handwriting was of considerable difficulty. They also reported difficulties organising coursework deadlines and managing multiple tasks simultaneously, such as writing tidily and quickly whilst listening to, and understanding, the lecture content. Given the contribution of handwriting speed to better outcomes in essays, lecture notes, and text notes [45], it is important to acknowledge the influence these skills have on successful academic learning, as they are positively associated with a better academic performance [45,46,47]. Additionally, we know that fine motor difficulties (which interfere with the automatised process of handwriting) negatively impact resources of working memory [40], which can also negatively impact academic performance [48,49,50]. Thus, any difficulties with handwriting could have a profound negative impact on university grades for students with DCD [8,48].

Using a social justice lens, this study aims to investigate the current lived experiences of students with DCD in Higher Education. Sixteen years after the very first study looking at the strengths and weaknesses of students with DCD [8], we aim to determine how they may have changed. In particular, we want to investigate the lived experiences of these students to provide rich data to help understand the nuances of DCD support that educational professionals may not be aware of or capture. Through this, we intend to provide recommendations for areas where universities could improve support for these students. We hypothesise that not much will have changed for students with DCD since Kirby et al. [8] first investigated this topic in 2008.

## 2. Methods

### 2.1. Research Design

An interpretivist approach with a phenomenological lens was utilised for this study. Semi-structured focus group interviewing was employed to understand the lived experiences of Higher Education students with DCD. We used focus group interviewing to allow participants to elaborate on and share their experiences of living with DCD whilst having a safe space to articulate their perspectives [51]. Two focus groups were employed to help students build on and interrogate the different experiences among them [52]. Focus groups were chosen (rather than individual interviews) as they facilitate interactions between the participants leading to more in-depth insights that may be elicited from one-to-one interviews. Additionally, the focus group is more efficient, gathering data from several participants at once.

### 2.2. Participants

Students with a diagnosis of DCD were invited to take part in an online focus group via email from the University of Surrey (UoS) DCD database or the Disability and Neurodiversity Services (DNS) at Royal Holloway University (RHUL). Out of 11 positive responses, 10 students (age 21–38 years, 6 women) were included as one student failed to attend the focus group. All participants had either achieved (*n* = 7) or were in the process of obtaining (*n* = 3) a Higher Education qualification. At both of these institutions, DCD (i.e., dyspraxia) is a condition that the disability team is aware of and supports. Students at both institutions normally have a tailored learning support agreement depending on their diagnosis.

#### Inclusion and Exclusion Criteria for the Focus Group

**Figure 1:**
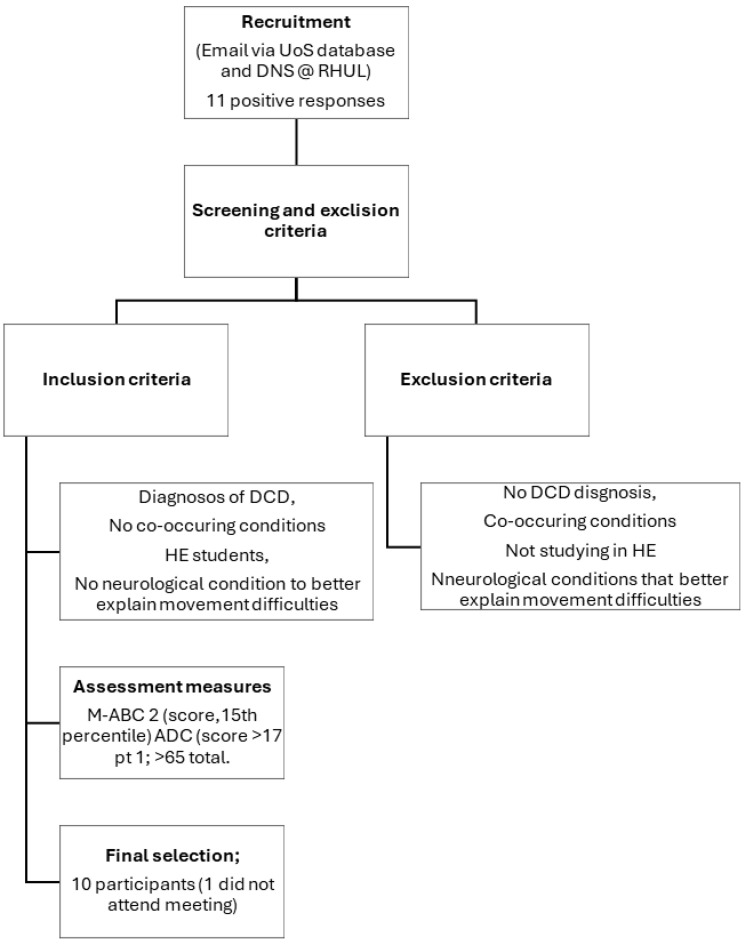
Flow chart summarizing the participant recruitment process.

All participants already had a diagnosis of DCD either from childhood or since starting their university education. Additionally, participants were screened using the four DSM-5 criteria for DCD [21] and UK guidelines for the assessment of adults with DCD [53]. Participants scoring below the 15th percentile (a strong indicator of poor motor skills and DCD; criterion 1) on the Movement Assessment Battery for Children-2 (M-ABC-2) [54], above 17 in Section 1 (an indication of movement difficulties in childhood; criterion 3), and above 65 in total (indication of poor motor skills in adulthood, impact on everyday living and probable DCD; criterion 2) on the Adult DCD checklist (ADC) [55] were included in the focus group meeting. As all participants were undertaking a university degree, we assumed a minimum level of average intelligence (criterion 4). These inclusion strategies reflect common practice in adult research in DCD [22]. Only participants without co-occurring disorders, e.g., ADHD, were invited to participate in this work. This strategy reduced the potential influence of more widely recognised and supported diagnoses. Participants were excluded from this study if they had a history of neurological or psychiatric disorders, had severe visual or auditory impairments or lack of a limb, or had a primary diagnosis of ADHD, dyslexia, ASD (see Figure 1 for flow chart summarizing the recruitment process and Table 1 for demographic details).

### 2.3. Materials

The Movement Assessment Battery for Children-2 (M-ABC-2) [54] was used to assess criterion 1 (movement difficulties) of the DSM-5 diagnostic criteria. The M-ABC-2 measures performance in balance, manual dexterity, and ball skills and is the most commonly used standardised test of motor skills for children. Given the lack of appropriate motor assessments in the UK for adults with DCD, the 11–16-year age band was used to identify adults with DCD. This strategy reflects common practice in research environments [23,39]. Individuals scoring below the 5th percentile demonstrate severe motor difficulties, and those scoring at or below the 15th percentile demonstrate moderate motor difficulties.

The Adult DCD/Dyspraxia Checklist (ADC) [55] was designed to address criterion 2 (movement issues interfere with everyday living) and 3 (DCD began in the early developmental period) of the DSM-5 and to help identify DCD in adulthood. The ADC is a comprehensive, self-report assessment that has previously been rigorously tested on individuals aged 17–42 years old [56]. A score of 17 or above in Section 1 is an indication of motor difficulties in childhood. An overall score of 56–65 or more for both sections is an indication of difficulties consistent with being ‘at risk’ of having DCD, and a score greater than 65 indicates ‘probable’ DCD [41]. To address criterion 4 and ensure that participants had an IQ in the typical range [32], cognitive ability was investigated through self-report of participants’ academic achievement. All students exceeded the minimum academic achievement requirement of 4 GCSEs.

### 2.4. Data Collection

The study was approved by the University of Surrey Ethics Committee (FHMS 20-21 043 EGA). Additionally, the participants were informed that they had the right to withdraw from the study during the interview at any point and that their transcript would be deleted immediately. If participants chose to withdraw from the study after the interview had taken place, they needed to let the researcher know within a month of the interview taking place. They were also told that they could stop or change the topic of the interview if it was too distressing for them.

Data were collected at two UK universities. Students from the university of Surrey (UoS) had previously participated in research and we used their existing ADC and M-ABC-2 scores to ensure the inclusion criteria were met. Five students responded to the email from Royal Holloway University of London (RHUL) and they were assessed on the M-ABC-2 and the ADC. However, only four of the students attended the focus group meeting. All the students tested met our inclusion criteria.

Qualitative data were collected via Microsoft Teams during two focus groups, one at the University of Surrey (*n* = 6, February 2022) and the other at Royal Holloway University of London (*n* = 4, April 2022). Note that the data analysis was performed once the two focus groups had happened. This was a suitable size as six to twelve participants is an adequate sample size to lead to meaningful data in an advisory group [57] and groups with larger numbers may not be appropriate when discussing sensitive topics [58]. To aid anonymity, participants were assigned numbers ranging from 1–10; participants 1–6 are from UoS and participants 7–10 are from RHUL. Both meetings lasted approximately one hour. The online format was chosen as it offers a cost-effective, convenient, and accessible option for collecting qualitative data. Participants attended the meeting from the comfort of their homes, which we hypothesise would aid honest and candid discussion. Additionally, the use of digital platforms such as Teams allowed the data to be recorded and transcribed easily. Whilst we acknowledge this method is not without issue, with a limit to the range of non-verbal communication we can observe. Overall, the method worked well as participants throughout the session appeared to be engaged and were providing meaningful and interactive discussions.

### 2.5. Reliability of the Analysis

To improve the dependability and credibility of the study [59], before conducting the interviews, we shared the initial questions with a member of the DCD community to determine the appropriateness of the questions. After refining the questions according to the feedback, the revised selection was finalised to be used in the focus group meetings.

#### Reflexivity

We adopted an interpretive, reflexive approach to the study to acknowledge that each researcher brings their own perspectives to the research and that this enhances, rather than detract from, the study [58]. Three of the researchers (JG, MM, and MI) reflected and discussed their own influence throughout the research to mitigate the effects of any intrinsic biases. This approach acknowledges the dynamic, iterative relationship between the data and the researcher [58] and facilitates a deep and dynamic evaluation of the data.

There were two interviewers in each of the sessions: one asked the questions and guided the meeting, and the other was there to support the proceedings, monitoring the online chat if any additional questions were asked by the participants. The advisory group was semi-structured with a small selection of pre-determined questions. The discussion was guided by the researchers, and the participants were encouraged to elaborate on their experiences, both positive and negative. In the case of the discussion going dry, the researchers asked another question on the list (e.g., about coping strategies they may have developed). Each participant was first asked to discuss their strengths and weaknesses as a student with DCD at university. The conversation then flowed organically and was participant-led; most of the other pre-determined questions were not needed as the participants mentioned a variety of topics (e.g., challenges on time management or navigation) when elaborating on the first two questions. De-briefings were held at the end of the session to allow group members to discuss any emotional distress. They were also asked if they had any questions regarding the study.

To address ethical issues unique to group approaches (lack of confidentiality and emotional distress caused by the over-disclosure of sensitive information during meetings), the advisory group members were asked not to discuss the content of meetings with individuals outside the group.

### 2.6. Data Analysis

The data from the two focus groups were qualitatively analysed once data collection was completed, using a reflexive, inductive, thematic analysis [58,60]. This strategy was employed to help understand the experiences of the students in the focus group and explore possible social injustices. An inductive approach allowed the data to drive the themes, whilst thematic analysis provided the flexibility to identify themes associated with participants’ experiences of support in Higher Education.

Both meetings were audio-recorded using the built-in features of Microsoft Teams. A verbatim transcription of the recorded dialogue was downloaded and anonymised for thematic analysis (TA) after the meetings finished. TA is frequently used as a method of analysis for qualitative research [58], but it is a subjective exercise that can be influenced by the researchers’ viewpoint [58]. Thus, the analysis needs to be conducted with rigour to ensure that themes are accurately and cohesively identified [61]. To ensure the rigour of our analysis, we adopted Braun and Clarke’s [58,60] six-step recommendations for thematic analysis. Firstly, Step 1, familiarising with the data; researchers JG and MI familiarised themselves independently with the data, listening to the recording, reading, and re-reading the transcripts to ensure that the transcription was accurate. Step 2, generating initial codes; initial codes were generated that identified meaningful data relevant to the experiences of students with DCD in Higher Education. Step 3, search for themes; codes were then grouped, based on the semantic meanings, into potential themes. Step 4, reviewing themes; a collaborative process ensued with three researchers (JG, MM, and MI) reviewing and refining the themes. Step 5, defining and naming themes; final overarching themes and subthemes were identified by ordering the codes and clarifying the researcher’s understanding to ensure that it fully represented the dataset. Specific examples from the transcribed data were also identified to be included in the narrative to provide an insightful account of participants’ experiences. Once these 5 steps were completed and the themes identified, we produced this report, thus completing Step 6.

In sum, this qualitative study employs reflexive thematic analysis to explore and understand the experiences of students with DCD in Higher Education. Through a rigorous and iterative analytical process, the study aims to uncover the challenges, coping strategies, and support mechanisms that impact their educational journey through Higher Education.

## 3. Results

**Figure 2:**
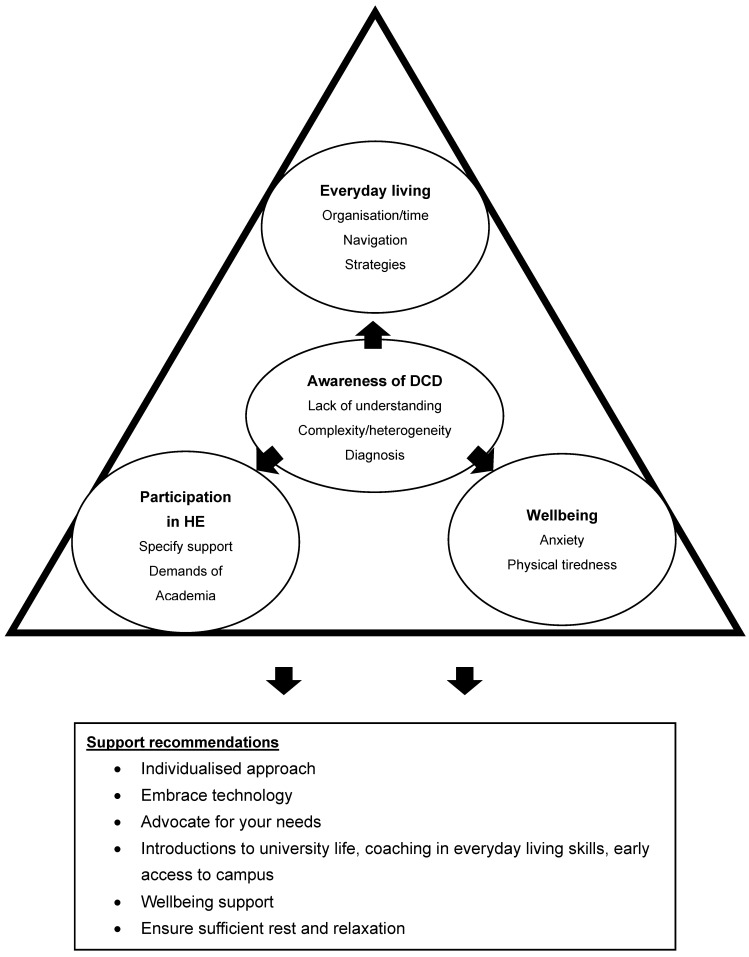
Themes and subthemes of the Higher Education experiences of students with DCD. HE = Higher education.

Whilst there was some variation in the two focus groups, four interrelated overall themes were generated from the data, offering insights into the current experiences in Higher Education for the participants. (1) ‘Awareness of DCD’ relates to the participant’s experiences and observations of the awareness of DCD among peers and staff in the Higher Education system. (2) ‘Participation in Higher Education for individuals with DCD’ relates to personal experiences whilst participating in studying in a Higher Education environment. (3) ‘Wellbeing’ relates to personal experiences of wellbeing issues related to the Higher Education environment, such as heightened anxiety and dealing with others’ perceptions. (4) ‘Everyday living’ relates to the physical toll that DCD has on many everyday living tasks. Please see Figure 2 for an overview of the themes and subthemes.

### 3.1. Awareness of DCD

In this theme, students reported varying levels of awareness about DCD among their lecturers, peers and even themselves, which led to inconsistent support. Three key factors affected support: a general lack of awareness of DCD, the complexity of DCD, and late DCD diagnosis.

#### 3.1.1. Lack of Understanding (Individual and Institutional)

Students felt that Higher Education Institutions (students, academics, and neurodiversity teams) had a lack of awareness and understanding of their DCD and an inability to fully contextualise the range of the everyday life skills that it affects.

“I’ve had a number of lecturers that are kind of not very understanding of that [DCD] at all.”

(Participant 9, RHUL)

There was also discussion about a lack of knowledge identifying which students in the cohort had DCD. These experiences led to situations that were far from ideal for a learning environment. In fact, one student (participant 1, UoS) even changed modules because the lack of understanding and support affected their learning. Additionally, the student felt a lack of willingness from teaching staff to adapt to their needs. This was not a singular example:

“I did have one difficulty with the teacher ’cause it was a one-to-one thing where she very clearly either didn’t know or didn’t think it was a thing [’cause I’ve come across that a few times before]… She set me an assignment, but didn’t give me very precise instructions, so I completely misinterpreted what she wanted, and it was a car crash. I did end up then changing module, so I wasn’t working with her anymore. But yeah, I it definitely felt like she hadn’t been briefed beforehand.”

(Participant 1, UoS)

Students further explained that they have experienced a general confusion about the attributes of DCD, where they felt that some people confused difficulties performing everyday tasks with a lack of intelligence. For example:

“And then she tried to explain to me the concept of heat. And I was like, I understand that fire cooks food. Yes, that’s not the problem, the problem is having multiple timings.”

(Participant 8, RHUL)

#### 3.1.2. Complexity/Heterogeneity

The complexity and heterogeneity of DCD present additional hurdles that can have a negative impact on understanding how DCD presents in an academic environment, further reducing awareness. The participants shared how their DCD manifested differently, even among those with a diagnosis of DCD, highlighting the need for an individualised approach to support. For example:

“I actually think that that is interesting that I’ve actually developed some of my more detailed motor skills. I’ve actually developed better as being a vet because I have to practice these things regularly, like over and over and over again.”

(Participant 5, UoS)

“I actually have almost the exact opposite problem to participant 5 and my gross motor skills are absolutely fine, but my fine motor skills are absolutely atrocious and calculating has always been a bit of a nightmare for me.”

(Participant 6, UoS)

#### 3.1.3. Diagnosis: Acceptance/Disclosure

Because of the lack of awareness of DCD, comments around acceptance/disclosure provided a novel insight into some of the issues associated with DCD disclosure. Some participants thought disclosure might take away individual autonomy or perceived control over the support offered. Whereby, disclosing your condition and accepting support necessarily means that you have to accept all the support offered even if it is not helpful. However, after engaging with the support teams, participants acknowledged the flexibility to choose appropriate support, as detailed below:

“… but I also appreciate that me bringing up my dyspraxia hasn’t meant that I’ve been railroaded into taking every single thing that the University has to offer that I could kind of at my own discretion choose what to pick up, what to use, and who to disclose it to that it hasn’t.”

(Participant 4, UoS)

The students also explained that they received their diagnosis at university, which helped their learning considerably. This diagnosis often leads to access to Disability Support Allowance (DSA), which. again, helps to alleviate some of the everyday issues that students with additional learning needs face. For example:

“I got diagnosed in my first year and I wouldn’t have got diagnosed if I had not been at University.”

(Participant 5, UoS)

“I have had a fairly positive experience at university. I received a diagnosis in my first university year but I wouldn’t have got a diagnosis otherwise. Following that I got support such as extra time in exams. […] I would like to say the DSA grant I got through student finance… was really helpful for me when I first got it… and yeah, beyond to like claim back for like printing expenses, having my own printer. And all the rest of it just took the stress level, not way down, but maybe took it down a notch that I didn’t have to worry about printing and the fact that you know organization was sometimes a problem. It meant that I did fill the DSA. I was really getting really good support.”

(Participant 1, UoS)

### 3.2. Participation in Higher Education for Students with DCD

#### 3.2.1. Specifying Correct Support

The second theme generated was Participation in Higher Education for DCD; within this theme, students expanded on the discussions detailed above, explaining how difficult it is to secure the appropriate support even though a diagnosis of DCD is available at the university. Nearly all the students in the focus group explained the difficulties of DCD being recognised as its own condition (with specific needs that should be tailored towards supporting the movement difficulties) to ensure that students with DCD can access the curriculum with equity. The students also explained that, while support was available, they felt it was often tailored to the needs of dyslexic students. For example:

“Yeah, I was gonna say probably very similar experience. I was also diagnosed at university but in my second year having had extra time and things and just being told I was probably dyslexic all through secondary schools but never quite fit the mould, so to speak of the dyslexic profile.”

(Participant 2, UoS)

“Where they have very generic support packages, it’s kind of tailoring that with the student I suppose and making sure that that’s what they need rather than just this is what we do for everyone with a specific learning difficulties or whatever.”

(Participant 9, RHUL)

Importantly, students felt that the support provided was sometimes inappropriate. However they also noted that they were often unclear about the specific support they needed, what worked for them, or what they should request. For example:

“The support is very “dyslexia focused” which isn’t always relevant for my diagnosis. I get overwhelmed audibly and visually in lecture halls and explaining that to lecturers is hard because I am not always aware what I need.”

(Participant 2, UoS)

“I think for me it’s just not knowing what I want.”

(Participant 3, UoS)

“I think it is sometimes assumed that you know exactly what you need. And I think that sometimes tricky as well.”

(Participant 9, RHUL)

“But when I got to high scores [this highlighted] the disconnect between what I was able to do verbally and on tests, and what I was able to do written. And that’s why I think the only thing they could think of at the time was extra time to give yourself that chance to slow down and make your writing vaguely legible. In terms of writing medical notes now my trust going over to electronics was an absolute godsend.”

(Participant 6, UoS)

However, while support may not always be tailored to students’ specific DCD needs, the students explained that the generic support of extra time provided in exam situations, normally associated with dyslexia, was helpful.

“The extra time that I would have in exams during my undergraduate was really essential because for me, just having the freedom of that security to know that I could take my time to formulate my ideas and actually put them down to paper was essential.”

(Participant 4, UoS)

#### 3.2.2. Additional Demands of Academia for Students with DCD

However, even with extra time in exams, handwriting and fine motor skills continue to be an issue for students with DCD in Higher Education even though access to computers and laptops is often available. Students shared that assessors often have difficulty reading their writing, with obvious negative implications for assessment. These issues can become particularly relevant when training in certain workplace environments that are heavily reliant upon the written word, such as the NHS (National Health Service). Such issues highlight not only the motor difficulties accompanying DCD, but also negative perceptions of others and low self-efficacy. For example:

“I’d get anxious when I was taking notes because I would be taking the notes and then I’d miss bits where I have been concentrating on what I’m writing.”

(Participant 9, RHUL)

“… I could find myself on placement with a [NHS; National Health System] trust (a unit within the National Health Services of England and Wales) that is 100% handwritten documentation… I was so worried beforehand that if I had had access to support beforehand, I think it would have just alleviated a lot of my worrying about it.”

(Participant 2, UoS, nursing student)

The need for extra time in other activities beyond writing also highlighted some issues that may be discipline-specific. For example, student’s experiences in differing disciplines (engineering and veterinary) highlighted more time is needed to practice newly acquired fine motor skills:

“well for me I do electronic engineering so I have to do a lot of hands-on soldering and stuff, and there was nothing extra for that, so we would have labs for the first two years of my study, and it’s just a one day a week—it’s like 3 h and then at the lab closes so you can’t do anything extra in the lunch gap. You can’t go in and out of those times, so there’s no extra time to like catch up ’cause I would be a lot slower than a lot of my peers and I would get marked down sometimes, but there was nothing they could do about it. That’s one thing I would say in some courses, maybe the support’s not there.”

(Participant 3, UoS, engineering student)

But for those who were provided with sufficient opportunity to practice these fine motor skills, there is a sense of achievement, for example:

“But I do find that fine motor skills I have had quite a lot of practice on and I feel like it’s developed better, maybe because I’ve had to focus on it and had to practice and had to get better.”

(Participant 5, UoS, veterinary student)

Aside from the fine motor skills, the participants shared their experiences in large lecture environments. They felt these settings can lead to sensory overload and make it difficult to organise and formulate a cohesive argument for in-class discussion:

“I find in like big lectures an there’s lots of background noise. I get really overwhelmed, and then I can’t like concentrate and I can’t hear, and I can’t focus because there’s so much going on and I can hear everything and like I just get a bit overwhelmed by all of that stimulation.”

(Participant 2, UoS)

“what I found myself to struggle with most going to uni is a lot of or some other lectures and seminars have this like participation mark. Which means you have to sort of like talk. And I really struggle with like form my ideas in my head out in full. So, when I speak out, sometimes it just sounds like gibberish.”

(Participant 10, RHUL)

Although universities did not always provide the specific tailored support that students with DCD felt they needed, several of the students found the university environment easier than school as they felt it offered them independence, flexibility, and autonomy to study at a pace that suited them. For example:

“I actually found the independence of university much better for me”… “I think that was the right balance of like independence and structure for me… having the freedom to like do at my own pace and kind of go a bit off script if I need too.”

(Participant 8, RHUL)

“I agree that overall university was definitely quite positive experience from my point of view.”

(Participant 1, UoS)

#### 3.2.3. Strategies for Academic Success

Whilst many students were unsure of the support they needed as they studied in Higher Education, they did develop some strategies to aid their academic success, such as through technology. Students embraced technology to support the motor demands of academic life as well as being organised and reflective about their own constraints. For example:

“There is the typing side, but there’s also the lack of ambiguity that you can put down in black and white in good text to speech. You actually do your work a lot quicker by using the technology properly. I think that’s what I noticed again coming at university second time with how much more focus on my electronic submission and almost minimizing the actual handwriting that you had to do.”

(Participant 6, UoS)

“I think one of the coping strategies I used to try and head off the fact that my writing is practically illegible and that was to really use speech detecting software.”

(Participant 6, UoS)

“Getting someone else to proofread your work if you can. Uh, because often even if I got loads of mistakes, I don’t spot them because my brain doesn’t really work like that.”

(Participant 10, RHUL)

Students also developed metacognitive and organising strategies to help them reduce cognitive load and stay on top of their university work, for example, making lists was a particularly popular strategy as detailed below:

“So you can make these lists and forget about them for the whole day and then but [I use] multiple alerts as well because one might go off and then sort of well, that’s fine, I’ll do that.”

(Participant 10, RHUL)

Being able to advocate for your needs was another strategy discussed; however, this was challenging for some, and the outcomes are not always positive, particularly when the students do not know what is available or what is needed. For example:

“And they’ve always been very good at kind of encouraging my work patterns. And if between supervisor meetings, it’s a case of not necessary, demanding that I have a written complete draft. More that I have an outline or something a bit with that sort of, that freedom of things it and that has been encouraging, and as far as other forms of support.”

(Participant 4, UoS)

“They’ve [Disability and Neurodiversity Services] heard [about] it [DCD] giving examples and things like that, but I don’t think it’s made a vast amount of difference because again, quite a lot of it is you having to ask for what you need, and if you don’t know what you need, it’s very difficult to say.”

(Participant 5, UoS)

Several participants commented on the value of printing off their notes, and, whilst this may not be so valid currently, as since COVID-19, most universities in the UK provide online recordings of lectures and assignments are submitted online, it might still be a useful strategy for some students with DCD.

“Having a printer in my own room rather than having to go into campus to print everything off was really really good. So, it meant that it just gave me like the extra like 15–10 min I needed for handing an essay and also mix it with one less thing I had to do ’cause I would have it when I went in to hand it in rather than having to print off and then hand it in.”

(Participant 1, UoS)

“I didn’t know whether this was done on all courses, but for my course in the first two years they actually printed all the notes off for us. And they give them to you in like booklets. And I would say I would have been screwed without them like ’cause I would have had the problem of panicking in the lecture. Can’t keep up, but we had most of the notes already. And I don’t know if all other courses did that, but I think they should ’cause I think it’s a really good thing.”

(Participant 3, UoS)

### 3.3. Everyday Living at University

#### 3.3.1. Organisation/Time Management

Students were also concerned about their time management for both their academic and everyday lives. Whilst there was no formal support for these activities, students developed their own strategies to help them. For example, they shared strategies to organise their time, ensuring timely arrival at lectures or social gatherings, as well as using alternative study formats, such as online lectures; for example:

“Remembering that I had to get there for the time rather than leave at the time. But yeah, a big thing for me.”

(Participant 4, UoS)

“I only know from my experience, but I missed a lot of lectures and stuff like I was all over the place, but a lot of the lecturers didn’t like recording the lectures, but now obviously it’s all online and I think the online format has massively benefited my studies.”

(Participant 3, UoS)

The students developed some useful strategies for managing everyday tasks, such as breaking tasks down into smaller steps, using timers as reminders, or completing tasks on quiet nights to avoid feeling overwhelmed. For example:

“I got into baking, but all the things I’ve baked, they’re very simple recipes and with baking I feel like it’s more as long as you get your work bench setup and you’ve got all your little ingredients and you’ve got your scales, you got all the things you need.”

(Participant 8, RHUL)

“I’d then do things like laundry/cleaning/food shop on Sat night when it’s quiet so no-one else is there—not sure if that’s a coping strategy or antisocial-ness.”

(Participant 8, RHUL)

[Talking about cooking] “I have a timer on my phone and I’m very precise. Like, I know this comes out this time and yeah, that’s OK for me”.

(Participant 7, RHUL)

The students also reported that the impact of DCD on tiredness negatively affected their completion of everyday skills. Cooking, shopping, and laundry were identified as particularly difficult, especially after a busy and tiring day at university as well as being able to keep the place tidy. For example:

“Cooking. I think that’s the only thing I really struggle with because it just takes me longer to, like, chop vegetables or something. And so, when I get home late at night, I just don’t wanna cook because it’s gonna take me like, an hour or something”. “And I don’t want to do that after a full day of lectures.”

(Participant 7, RHUL)

“I can cook, it just takes me ages, and also I have trouble with the timings of like when to put things in, when to take them out…”

(Participant 9, RHUL)

“Laundry was like a no go for me… I think I have so many clothes. Like, really cheap clothes. Just because I didn’t like going to the laundromat and doing it, something that people don’t really understand. I think people underestimate life skills, especially after you’ve had a full day at work, and you get in and you’re just not going to be in the mood to cook or do laundry or anything.”

(Participant 8, RHUL)

“But the thing I have the most trouble with is the cleaning aspect. Because there is mess everywhere because you use everything, and I don’t clean up in the way that is too stressful.”

(Participant 10, RHUL)

Hence, these everyday chores are an important consideration when planning academic support for individuals with DCD, yet it is often overlooked within the context of academia.

#### 3.3.2. Navigation

Several students reported navigational difficulties, explaining that finding their way around a new university campus was challenging. Whilst maps are often available, they are also difficult for a person with DCD to contextualise and can result in arriving late for lectures and impacting academic engagement and anxiety. For example:

“They always like spread the classes out into random different buildings… ’cause you have to find a new room and then next week you’re in another room and it’s just it’s a little bit irritating.”

(Participant 7, RHUL)

“For someone who has anxiety anyway, so that’s part of my own issue. But when you’re then stressed ‘cause you can’t find somewhere and then you’re anxious enough about going to lecture, it is just kind of, I… didn’t realize how much of an impact it had had until you asked that question.”

(Participant 2, UoS)

There were no specific support activities to aid navigation, but students shared strategies to support their navigational difficulties:

“So… if I was asked, now find your way to this part of the university that you’ve never gone to…, I probably would go OK, I will have to take a day or so to just make sure that my I know what my route is, … and so it feels like I need to be very targeted with where I go to find my way”… “And that means that I that’s probably been my coping mechanism that… I do a good job of highlighting which parts of the university are the most important ones, and if for whatever reason there’s an event or circumstance that’s taking you away from your usual area of interest, that’s where I think sometimes the issues can arise.”

(Participant 4, UoS)

“I’m generally OK with navigation, but I’d say with the maps and things, I like to familiarize myself with where I’m going beforehand… I find Google Maps very accessible, whereas later trying to load the university maps…”

(Participant 9, RHUL)

“the [campus] tour already helps...there are so many different events on campus, and they put them in like all the different buildings I think to make you go to all the different buildings and get experience of them and that really helped.”

(Participant 8, RHUL)

### 3.4. Wellbeing

#### 3.4.1. Anxiety

Students discussed their general wellbeing with respect to their DCD diagnosis and studying at universities. In particular, they expressed how their DCD diagnosis heightened their anxiety about how others might perceive them when submitting work or being punctual, for example:

“So, like my first placement, wasn’t until right at the end of first year and I spent all of first year really, really worried about so many different elements and they seemed really silly things to be worried about. So, for me my handwriting is appalling. And I knew I was going to be expected to be writing a medical note, but other health care professionals to read. And I was like I can’t even read my own handwriting, and I spent like months like worrying and trying to practice my handwriting beforehand.”

(Participant 2, UoS)

Some students, however, were taking positive steps in dealing with their wellbeing, such as through counselling and being aware of methods and strategies to support their anxiety. However, this did not appear to be a universal approach within the university to support DCD, but rather an individual choice:

“I think that I and a lot of people have dealt with mental health issues of anxiety and things like that… so it’s sort of all part of how your head makes sense of things, and I think that when I’ve discussed this with the counselling services, they have always made me aware of what methods and avenues of support there are…”.

(Participant 4, UoS)

#### 3.4.2. Physical Tiredness

Physical tiredness was a common theme for the students, as they explained how even just preparing for the day (dressing, food prep, and time management) left them physically tired, before any academic interactions took place:

“I feel tired of all the time, and I don’t know if it’s dyspraxia or not, but yeah, I’d say tiredness and also possibly slightly more stressed than our peers at times.”

(Participant 9, RHUL)

“I definitely feel tired, but I think for me, it’s mostly mornings like I’m not a morning person anyway, but I think it’s the concept that you have so much to do, just to even leave the house”… “I feel quite tired and stressed in the mornings, definitely.”

(Participant 8, RHUL)

This area of impact is rarely discussed, but has serious implications for accessing the educational curriculum equitably.

## 4. Discussion

This research investigated the lived experience of students with DCD in the UK Higher Education system using focus group interviews. The research extended Kirby et al.’s [8,29] and Sumner et al.’s [9] studies, updating the post-pandemic lived experiences of students with DCD in a country that has a legal duty to provide equitable access to education. Four main areas were identified as priorities to improve experiences in higher education for individuals with DCD: (1) developing further awareness of DCD amongst educational and disability professionals, (2) identifying and providing specific and appropriate support for students with DCD, (3) supporting students’ wellbeing/anxiety, and (4) supporting students’ everyday living skills.

Through this study, the overriding message from the students is that the formal support provided by their Higher Education provider is helpful, albeit often targeted at dyslexia rather than DCD. Several of the students in this study were diagnosed with DCD at university, and they were positive about this experience, the academic benefits, and the support they received from their Disability and Neurodiversity services. However, the support provided was targeted towards study adjustments, particularly within the formal classroom environment. An important omission of current support within the academic environment is recognising the disproportionate impact that the many activities of everyday living has on individuals with DCD. Many of the students discussed the negative impact that tiredness has on both personal care and academic achievement. This needs to be an area of focus in the future, that is, creating support mechanisms for neurodivergent students who are managing a high level of additional constraints often before they even arrive at the university campus.

### 4.1. Higher Education Institutions and Their Students Do Not Understand DCD

One of the clearest messages from this focus group is that, whilst Higher Education Disability and Neurodiversity Services are good at diagnosing DCD, and willing to offer support, the support does not appear to be specific and suited for individualised needs. The reasons for this are complex; on the one hand, the students themselves are often not confident in their rights to articulate the support they need. On the other hand, because DCD is itself a complex condition [33,62] and presentation can vary, there may not be a neat solution. However, whilst the one-size-fits-all approach is not perfect, for some students, it has provided the support that they otherwise would not have had access to. Furthermore, the solutions available from the Disability and Neurodiversity Services do not extend to the issues identified within this study, in particular supporting everyday skills and sensory issues.

Most of our focus groups members, however, shared that their experience at university was positive, supporting previous findings by Sumner et al. [9]. However, this positivity could also originate from the students with DCD feeling their needs have finally been acknowledged, as nearly all the participants were diagnosed at university. Alternatively, it could highlight the resilience and problem-solving skills of these students as they have already developed their own academic and everyday living strategies, having successfully accessed Higher Education in the first place. Whether they are representative of the wider DCD population is beyond the remit of this work, but, given the challenges that students with DCD face, it may be that only exceptional students are currently accessing higher education. This clearly needs to change.

In addition to a lack of understanding from Higher Education Institutions, the lack of awareness in the general population of students, university teaching staff, and even clinicians [63], makes the university experience much more complex for students with DCD. If fellow students knew more about DCD, they would not make negative comments such as those mentioned in the focus group, they would be more understanding, and a more inclusive environment would emerge. One major issue for students with DCD is that they must repeatedly explain their condition or face being misunderstood. This constant worry that a lecturer would not understand written notes, housemates would find them messy and seemingly disrespectful, or that someone would think they lack intelligence because of how their DCD expresses itself is a source of heightened anxiety for them. Knowing that the transition to Higher Education is difficult for any student, there is an urgent need to educate people and raise awareness about DCD and the impact it has on academic study.

Heterogeneity was also mentioned by several students, with some reporting severe gross motor difficulties and others reporting only fine motor difficulties. These comments reflect the current discussion in DCD research aiming to categorise the motor difficulties into subgroups. Lust et al. [64] argued for four main subtypes, which include (1) all motor skills reduced; (2) motor skills reduced, except for gross motor ones; (3) gross motor/balance difficulties; and (4) fine motor difficulties. It could be argued that Higher Education Institutions are not clear about the different presentations of DCD, which presents another barrier to improving awareness and tailoring appropriate support, which may need to be based on these categories in the future. However, this research is in the early stages and much more work is needed to fully evaluate the impact these subgroups have on academic achievement, wellbeing, and everyday living skills.

Heterogeneity is not only apparent in the diagnostic process of DCD, but also in the mixed levels of understanding within and between university faculties. We have two examples from different specialisms mentioned in the focus groups, that highlight how students with DCD generally take longer to learn new motor skills (as discussed by the engineering and veterinary students). However, differing attitudes and support from instructors can directly impact student academic success. If students with DCD who take courses with a motor component are not allowed extra time for practice (as was the case for the engineering student), this can negatively affect not only their academic success but self-esteem and perceptions of ability (self- and others’). Not only does this negatively impact the students’ experience, but students with DCD are consciously making degree course choices based on motor content rather than an equitable choice based on ability and interest [8]. It is thus crucial to offer extra time for students with DCD beyond exams, such as in labs; one could even argue that extensions for assignments should be standard for these students, given the additional time they need to access academic materials (such as rewatching lecture recordings to negate issues with handwriting or sensory issues in lecture theatres), organise their ideas and write their assignments compared to their neurotypical peers.

One further barrier to awareness of DCD is the perceived stigma associated with disclosure; individuals can feel embarrassed about their condition and fear that others’ will judge them as less intelligent, and/or lacking in credibility [65]. Additionally, Clouder et al. [66] argue that, whilst higher education institutions have good intentions and available technologies to support students with specific learning difficulties, concerns about labelling and stigmatisation are creating a mismatch. If students do not have the confidence (or, in the case of DCD, knowledge that they can access support) to ask for help, this further exacerbates the issues faced by students with DCD who may not even know they have the condition and that they are legally entitled under the Equality Act [4] to ask for support.

There are many overlapping themes and subthemes in these data. For example, lack of awareness of how to support DCD, from the university Disability and Neurodiversity Service teams, teaching staff, and students, created a far from optimal learning environment. Students with DCD are often left feeling excluded and overwhelmed and, in many cases, struggling to keep up. These negative influences can lead to increased anxiety, which in turn affects cognition, engagement, and quality of work [67,68]. Many of the issues that students with DCD in Higher Education face lead to a failure in support of their equitable access to the curriculum. This may have negative repercussions on their final degree grade, reducing options for future study or employment, another area of disparity for adults with DCD [25]. Further to the scope of this work, the lack of awareness of DCD is a worldwide issue [69] that needs to be addressed in the same way it has been conducted for more known specific learning difficulties, such as ASD or ADHD.

### 4.2. Secondary Implications of DCD Negatively Affect Academic Success

Several comments highlighted that the motor coordination difficulties are often accompanied with secondary issues that impact the efficiency with which individuals with DCD can engage with academia as well as many everyday activities. In the academic environment, it is particularly important to recognise the interconnected relationship between the motor, executive function, and attentional systems [28,34,70]. This relationship becomes highly relevant when considering the requirements of notetaking during a lecture. The students explained how difficult it is to adequately allocate cognitive resources when the motor demands of writing (or typing) notes are so high [8]. Several mentioned relief that the recording of lectures was now standardised in most universities since the COVID-19 pandemic, enabling them to re-visit the lecture in their own time. More research is clearly needed in this area to investigate the best ways to support students with DCD in Higher Education and disseminate the solutions to Disability and Neurodiversity Service teams in all UK universities.

For many adults with DCD, the motoric difficulties often become secondary to concerns about executive function, daily living activities, and learning new routines [22,71]. The students discussed the additional effort needed with planning and prioritising their work to ensure it is completed in a timely manner, several students commented that it often takes them longer to complete tasks compared to their neurotypical peers. These findings reflect research in DCD identifying that executive functional difficulties with organisation, planning, and speed of processing are important considerations for adults with DCD [22]. However, an area not mentioned in previous research [8,9,29] is the physical toll that DCD has on the individuals, which often results in little attention being paid to important self-care activities (such as preparing and eating nutritious meals) or engaging socially with friends. Additionally, several of the students explained that they manage their everyday living tasks, for example, in the supermarket or laundry, to avoid busy environments. Whilst this is an excellent strategy to help navigate some of the constraints associated with DCD, it may have an additional effect of reducing opportunities for social participation. If students are doing their shopping/laundry on a Saturday night when the shops/laundry is quiet, they may not then be taking part in social activities.

Educational resources are essential for students with DCD to succeed in Higher Education, but daily living strategies are also imperative. Poor postural control and fine motor skills affect the efficiency of self-maintenance activities (i.e., personal hygiene, dressing, and eating behaviour) in children with DCD [72,73]. Stronger daily living skills predict more positive adult outcomes, including better perceived wellbeing and higher education rates, employment, and independent living. For these reasons interventions to support daily living skills should now be prioritised in DCD; similar to intervention in ASD [74,75].Approaches like Cognitive Orientation to daily Occupational Performance (CO-OP), which teach strategies to acquire personally meaningful functional skills, help children with DCD in improving their daily living skills (e.g., [76,77,78]). From the present work, it is clear that DCD students still struggle with their occupational experience and household chores [79]. However, they receive no help in these areas and have to be more creative, tenacious, and resilient and come up with strategies themselves. Transition to Higher Education is stressful; students sometimes leave their family home for the first time and have to manage new tasks, like cooking and laundry, alongside their academic endeavours. Thus, additional support for these activities will benefit all students, both with and without DCD. However, students with DCD face an additional toll compared to their neurotypical peers given their difficulties in motor coordination, multitasking and time management, skills that are crucial in daily living. The adverse consequences can be very important, leading to frustration and anxiety and a globally reduced mental and emotional wellbeing [79].

Another concerning, but not unexpected, area of discussion was the prevalence of wellbeing issues in the group. It is well documented that individuals with DCD tend to have higher rates of anxiety and depression than their neurotypical peers [25,80,81], and almost all participants discussed some issues with anxiety. Causality is difficult to pinpoint, but from the discussions in the focus group, it appears that, in a Higher Education environment, many of the anxieties emanate either from personal constraints associated with DCD (movement, planning, and time management difficulties) or environmental constraints, such as difficulties finding appropriate lecture theatres, participating in class activities, or keeping up with the motor demands of the course. Whilst it is beyond the remit of this study to establish whether the heightened anxiety in DCD is a reflection of a predisposition to anxiety for this population, or a result of difficulties in everyday living, it is appropriate to mention the Environmental Stress Hypothesis here to help unpick the interconnected relationship between personal constraints and environmental ‘stressors’ that have been argued to contribute to higher rates of anxiety in DCD [82,83]. Cairney and colleagues argue that the additional challenges faced by individuals with uncoordinated movement when going about their everyday lives creates a ‘hostile’ environment that leads to heightened anxiety. These ‘additional challenges’ are not limited to the physical constraints of the individual or the environment but can also include the negative perceptions of others and feelings of low self-efficacy [80,84].

The students commented that wellbeing support was available and easy to access and that mentors were able to signpost avenues for support. However, for wellbeing mentors to fully understand the issues of students with DCD, they also need an understanding of the stressors for this population, which may be different to those of the general public. For example, Harris et al. [80] argue that adults with DCD have higher levels of general and movement-specific anxiety. Given the research [25,80,81] and conversations with the focus group, it seems highly relevant that mentors should be aware of this added layer of complexity to be able to appropriately offer tailored support. Increasing knowledge and understanding of this condition will go a long way to reduce the effects of the environmental ‘hostility’ students with DCD face in Higher Education.

Difficulties with navigation was another topic discussed by many of the students and described in the literature [85]. We argue that it would therefore be of benefit to these students if they were offered access to early campus visits that are often recommended for students with ASD. This strategy helps the new student to acclimatise to the campus environment with minimal distractions, often under the direction of student supporters who can provide tips for finding the best places on campus as well as sharing the best routes. However, here, lack of knowledge again impacts this intervention, as many students with DCD are not diagnosed until they reach university, and so, this strategy would not be available until after the semester starts, which then negates the advantage of a quiet and student-free environment in which to acclimatise.

### 4.3. Support Available for Students with DCD in Higher Education

A number of the participants were diagnosed during university, which indicates that the Disability and Neurodiversity Services have an essential role in helping students with Specific Learning Disorders to access the curriculum. Every participant from our advisory groups indeed received some support through the Disability and Neurodiversity Services from their university; however, this support was not always adequate. The offered support packages were often very similar to those offered to students with dyslexia, reiterating the findings from Sumner et al. [9] and Kirby et al. [8].

Besides the need to better tailor current packages to DCD, a common thread running through the discussions was that often students did not know what to ask for in terms of support. At this stage, we are not sure whether this is because of a lack of understanding of the resources available, the tailoring of resources towards other conditions, or whether our participants, having been mostly diagnosed at university, have already created strategies to cope successfully despite their difficulties. However, something needs to change quickly if universities are going to meet their legal obligations in the Equality Act [4], which prohibits discrimination in Higher Education on the grounds of nine protected characteristics, including Disability. The act covers areas such as admissions, education and training, student services, and facilities, and educational institutions are required to make reasonable adjustments to accommodate the needs of disabled students and must actively work to prevent discrimination. A poignant comment highlighted the need to ensure that autonomy is maintained throughout the support process to allow the individual to personalise their support and not feel ‘railroaded’ into accepting all that is on offer, which may become superfluous and overwhelming.

Given the heterogeneous nature of DCD, discussions about strategies for success were diverse, but there was some consensus. For example, most of the participants mentioned difficulties with time management and planning, reflecting research previously mentioned [22,71]. Technology is an important addition to our battery of support for students and this could be easily adapted to provide notifications, alarms, or signposts for students with DCD. Furthermore, these technological interventions could well be useful to many students, particularly in their first year of study when they are stepping onto a very steep learning curve. It is also acknowledged that interventions designed to support students with additional learning needs can often benefit the general population too.

Among the support that was most effective and should be offered to students with DCD was the extra time during exams, which allows them to take more breaks, but also to take the time to formulate their ideas better. Using a computer was also helpful for students for whom handwriting was an issue, but some students raised the point that handwriting the notes helped them learn the content. There should thus be some flexibility in the support offered and students should be provided with the possibility to test different support systems before committing to one. One thing for sure is that, if a student types their lecture notes because it helps them to take notes in a timely fashion during the delivery of this lecture, but needs to handwrite the note once at home to learn them, this is extra work that can be an additional toll for students with DCD. Additionally, recent work showed that handwriting and keyboarding share processes, and so, using a laptop might not be a fitting solution for every student with handwriting difficulties [86], reinforcing the need to an individualised and systematic approach [87].

Given the range of topics discussed in the focus groups, the overall picture illustrates the symbiotic relationship between the constraints of the individual (motor, cognitive, and wellbeing), the environment surrounding them (educational, social, and domestic), and the successful completion of the academic task. Newell’s constraints theory [88] works well to describe this relationship. Developed in 1986 and refined by Haywood and Getchell in 2009 [89] to explain motor development, this model is also applicable to the learning environment for those with DCD. Without a greater fluidity of information flow between all elements in this triad (individual, environment, and task), success in academia will be limited and frustrating for many students with DCD.

The overriding message from the students is that the support currently provided is helpful, albeit targeted at dyslexia rather than DCD. However, specialised support is much needed, but currently, the exact support is elusive, both in terms of selecting appropriate choices from a battery of options and, more importantly, knowing what the students need. However, a consensus from the focus group was that an individualised approach to assessment was needed, supporting a previous argument by Sumner et al. [8]. However, the assessor would need to look beyond the remit of dyslexia support to enable appropriate tailored support. Whilst more research is needed to specify exactly what support is most needed for this population, a list of support options available would be a start and would allow the student to specifically target their support to their needs and the motor demands of the chosen course. Support could include laptops, extra time (in exams, assessments, labs, etc.), early campus visits, and student buddies. Offering these options also helps with undiagnosed, multiple co-occurring disorders, providing the student with autonomy over their learning.

### 4.4. Limitations

There are some limitations to these data as the sample size was, by necessity, small and used data from two UK universities. Thus, the findings cannot always be generalised to the relevant population. However, the data we collected were detailed and the participants were able to fully elaborate on their answers. Additionally, the study benefitted from data collection in two separate focus groups at different universities (albeit both were in the Southeast of England). There was some difference in themes from each meeting, but overall, the insights gathered from the two meetings enhanced data triangulation, improving the reliability and validity of the findings.

One of the challenges of running two independent sessions is that the direction of the discussion followed different trajectories as the conversations progressed. This meant that both sessions did not cover all the same topics. However, the data provided were richer and more diverse and probably more representative of the experiences of students with DCD in comparison to running one meeting.

Following on from this, we received some very specific examples of difficulties with accessing parts of the curriculum that need a high level of motoric proficiency, which again might not generalise to the wider DCD community. However, we would argue that the examples highlight an important point, that DCD has an impact not only on programme choice, as argued by Kirby et al. [8], but also on access to the curriculum once the chosen course has commenced. It is likely that the type of disability model used to issue support may also affect the way support is provided and perceived. In this research, support was provided on a combination of a diagnosis and need-based. Further work is needed to determine how these models affect the perceptions of support.

Our final limitation was that there were no minority issues discussed in the meetings. This is an area in need of future research as we recognise that student identities (e.g., gender, ethnicity, and class) and their intersectionality’s may affect the prevalence and success of students with DCD in Higher Education.

### 4.5. Future Work

The findings from this research highlight several areas that still need further investigation. For example, one key area for exploration is the recognition and support of DCD in HE, particularly given the lack of specific data on DCD in the Higher Education Statistics Agency (HESA) reports. This gap in statistical data is a critical area of future research interest, as currently it limits the understanding of how DCD is specifically recognised and accommodated within HE institutions. Another area of future research should focus on how Higher Education institutions can better guide students with DCD in identifying their needs and ensuring they have access to the appropriate resources. Best practices for supporting students with DCD at the institutional level, including the role of educators in fostering academic success, are crucial topics for future investigation. Research in this area could lead to actionable recommendations for institutions to raise awareness among educators and improve the overall student experience for individuals with DCD. Finally, future work could explore the most appropriate types of technology used by students with DCD to support their academic success, as well as the benefits and limitations of these tools. Findings from this work would help identify new strategies and tools needed to improve the learning outcomes for students with DCD.

## 5. Conclusions

This research demonstrates that, even within a legal framework, and high prevalence of DCD, there is a limited understanding of the complexities of DCD in the Higher Education learning environment. Therefore, to improve the experiences and equitable accessibility of higher education to students with DCD, our investigations suggest that there is a need to (1) raise awareness of DCD within universities, (2) provide differential support for students with DCD in their studies, (3) provide support on everyday living tasks such as laundry and cooking, and (4) support their wellbeing. This work aims to facilitate this by (1) raising awareness of common themes relevant to students with DCD and (2) sharing strategies for success identified by the students for both academic and everyday living tasks. The information gleaned from this work can inform the development of resources that are most beneficial to students with DCD, but the reach of the benefits may extend to all students.

The findings from this research provide an excellent opportunity to enhance teaching quality by pioneering evidence-based pedagogic practices for students with DCD and enhancing the accessibility of educational resources for this population. Building on this first step, the next step would be to develop resources for students with DCD. Ultimately, this work will have a real-world impact by improving the skills, capabilities, and academic outcomes of students with DCD. It will also help to address current legislative requirements (Equality Act [4]) for institutions to be proactive in their efforts to develop inclusive learning environments, signposting a pathway to better employment opportunities.

The evidence presented in this paper highlights that there is still much work to be conducted to fully enable equal access to Higher Education for students with DCD. An initial focus on raising awareness at an institutional level should be enhanced by working projects (recruiting disability and neurodiversity teams, educators, and students with DCD) to confirm that appropriate measures and technologies are tested, verified, and offered to students with DCD. The information gathered from these interactions could inform the development of checklists to encourage a more individualised approach to supporting students with DCD, whereby they can specify their own educational support needs. This process will additionally ensure that Higher Education institutions can establish best practices and better guide students in identifying their specific needs to address some of the current inequalities facing this student population. Importantly, strategies learnt at the Higher Education level may also be appropriate for students with DCD studying at secondary and even primary levels.

Given the importance of educational qualifications to enter the workplace, and the positive contribution that employment can contribute to people’s health and quality of life, this is an important and novel addition to the literature, and one that is long overdue.

## Figures and Tables

**Table 1:** Demographics for focus group attendees.

Demographic Information for Focus Groups		
Gender	Man	4
	Woman	6
	Total	10
Age (years)	Range	21–38
	Mean	25.8
	SD	3.9
Education	* HE Qualification (obtained)	7
	* HE Qualification (studying)	3
DCD Assessment	* M-ABC-2 (Mean percentile)	2.9 (0.1–9)
	* ADC Section 1	25 (24–26)
	* ADC total	85.3 (68–97)

* HE = Higher education; M-ABC = Movement Assessment Battery for Children (M-ABC-2) [52]; ADC = Adult DCD checklist [53].

## Data Availability

The original contributions presented in the study are included in the article, further inquiries can be directed to the corresponding author/s.
